# Investigation of TID-induced capacitance variation in GaAs edge-lift capacitors and its effect on RF impedance matching

**DOI:** 10.1038/s41598-026-42919-9

**Published:** 2026-03-04

**Authors:** Min-Su Kim, Hyeon Jun Hwang, Chang Goo Kang, Jeong Min Park

**Affiliations:** 1 Department of Semiconductor Engineering, Mokpo National University, Jeonnam, 58554 Korea; 2 Advanced Radiation Technology Institute, KAERI, Daejeon, 56212 Jeonbuk Korea

**Keywords:** Total ionizing dose (TID), GaAs MMIC passive components, Edge-lift capacitor (ELC), Dielectric permittivity, Electromagnetic (EM) modeling, Impedance matching network, Engineering, Materials science, Physics

## Abstract

This paper investigates the total ionizing dose response of passive components fabricated in a commercial GaAs process, with a focus on dose-dependent capacitance variation in fringe-field–dominant structures. Measurements indicate that the edge-lift capacitor exhibits the highest total ionizing dose(TID) sensitivity, with its effective capacitance increasing from 7.65 pF to 22.96 pF after 300 krad(Si) irradiation at 10 GHz. This behavior is consistently reproduced in electromagnetic simulations by increasing the relative permittivity of the SiN dielectric from its nominal value of 6.9 to 8.5, enabling a radiation-equivalent dielectric modeling approach. When applied to an RF amplifier matching network, the TID-induced capacitance variation shifts the input impedance from 43.75 + j27.15 Ω to 33.8 + j5.35 Ω, accompanied by degradation in gain and noise performance. These results demonstrate that TID-induced dielectric property changes in GaAs passive components can be effectively captured using radiation-equivalent electromagnetic modeling and can significantly impact RF circuit performance.

## Introduction

Spaceborne communication systems are continuously exposed to accumulated total ionizing dose (TID) in low-Earth-orbit and must maintain stable Radio Frequency (RF) operation under long-term radiation environments^1,2^. Ionizing radiation can induce trapped charges, modify dielectric polarization, and form interface states in semiconductor and dielectric layers^3^, which may translate into parameter variations in RF circuits.

Prior studies on total-ionizing-dose effects have predominantly focused on active semiconductor devices, including GaAs FETs, GaN HEMTs, SiC MOSFETs, and advanced CMOS or FinFET technologies^4–7^. Reported degradation mechanisms include threshold-voltage shifts, mobility reduction, increased leakage current, and changes in on-resistance caused by radiation-induced charge trapping and defect generation^8,9^. While these degradations critically affect active-device performance, the impedance conditions required for optimal RF operation can also be affected by radiation-induced variations in passive components.

In Gallium Arsenide (GaAs) monolithic microwave integrated circuit (MMIC) design flows, impedance matching networks rely on integrated inductors and capacitors whose frequency-dependent characteristics are represented by electromagnetic (EM) models provided in the process design kit (PDK). These models assume fixed material properties and therefore do not account for radiation-induced changes in dielectric permittivity or loss. Consequently, dose-dependent variations in inductance or effective capacitance are not predicted during circuit simulation, even though such changes may distort the impedance trajectory of a matching network or shift its effective operating frequency. Understanding how radiation-induced dielectric-property variations affect fringe-field–dominant passive components, and how such variations propagate into circuit-level impedance behavior, therefore remains an insufficiently explored issue in GaAs MMIC technologies.

The radiation sensitivity of RF passive components can be strongly structure-dependent. Spiral inductors and thin-film resistors are largely governed by metal geometry or bulk resistive properties, with relatively limited contribution from fringing electric fields interacting with surrounding dielectrics, and thus exhibit small parameter variation under TID exposure^10,11^. In contrast, edge-lift capacitors (ELCs) are fringe-field-dominant structures in which electric fields are strongly concentrated near the lifted metal edge, making the effective capacitance more susceptible to changes in the local dielectric environment[12.^13^].

Here, we investigate the TID response of GaAs edge-lift capacitors and quantify dose-dependent capacitance variation using de-embedded RF measurements. Spiral inductors fabricated under identical process and irradiation conditions are evaluated as a comparative reference. Furthermore, radiation-equivalent EM modeling based on dielectric-permittivity modification is employed to interpret the measured behavior at the device level and to assess how passive-device parameter variation induced by ionizing radiation can propagate into circuit-level impedance detuning in a representative RF matching network.

## Results

### TID-dependent capacitance variation of the edge-lift capacitors

The effective capacitance of the edge-lift capacitor (ELC) was extracted from the de-embedded input admittance based on the imaginary component of the measured input admittance. Although the S-parameters were measured up to 20 GHz, the capacitance was plotted only up to 10 GHz because the self-resonant frequency (SRF) of the ELC occurred near 11 GHz, above which the extracted capacitance becomes unreliable due to resonance effects.

**Fig. 1:**
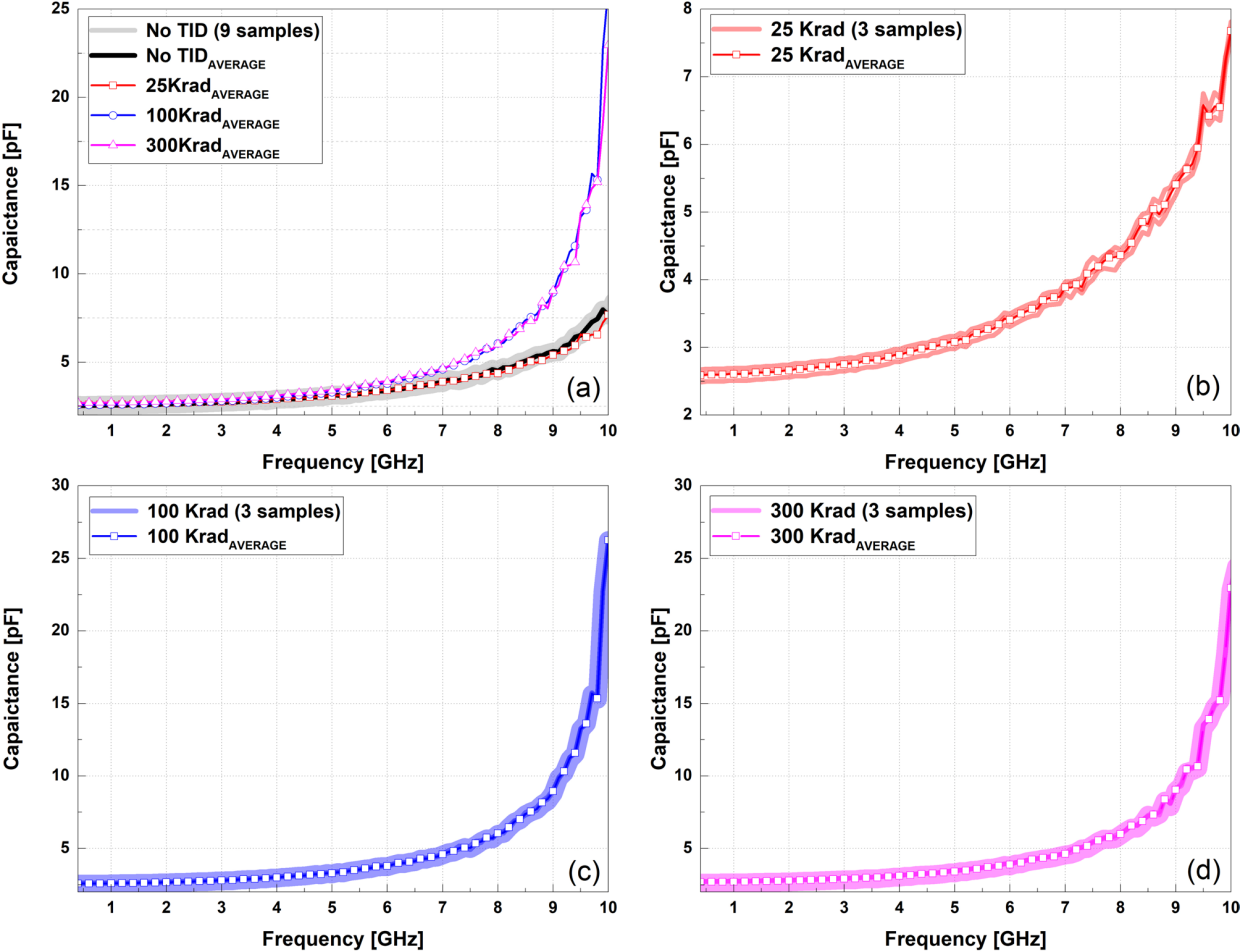
Measured effective capacitance of the ELC for nine samples under four TID conditions. (**a**) 0 krad samples with the mean values at 25, 100, and 300 krad. (**b**) 25 krad. (**c**) 100 krad. (**d**) 300 krad.

Figure 1 summarizes the capacitance measured from 9 samples under the four total-ionizing-dose conditions (no TID, 25, 100, and 300 krad(Si)). Subfigure (a) shows the frequency-dependent capacitance of the 9 samples at 0 krad(no TID), along with the mean capacitance values obtained at 25, 100, and 300 krad(Si). Subfigures (b)–(d) present the three samples and the corresponding mean value for 25, 100, and 300 krad(Si), respectively. Within the plotted frequency range, the mean capacitance increases progressively with accumulated dose, while the sample-to-sample variation remains smaller than the dose-dependent shift. This indicates that the effective capacitance variation is dominated by accumulated radiation effects rather than measurement dispersion. Representative capacitance values at 2 GHz and 10 GHz are summarized in Table 1.

**Table 1:** Extracted mean capacitance and sample variation at a representative frequency.

Dose (Krad)	Mean C_eff_ @2GHz [pF]	Std. Dev. σ @2GHz	Mean C_eff_ @10GHz [pF]	Std. Dev. σ @10GHz
no TID	2.62	0.04	7.65	0.07
25	2.66	0.09	7.67	0.12
100	2.66	0.04	26.26	0.04
300	2.77	0.06	22.96	0.14

### Equivalent series resistance of the edge-lift capacitor

The equivalent series resistance (ESR) of the edge-lift capacitor was extracted from the real part of the de-embedded input impedance.

**Fig. 2:**
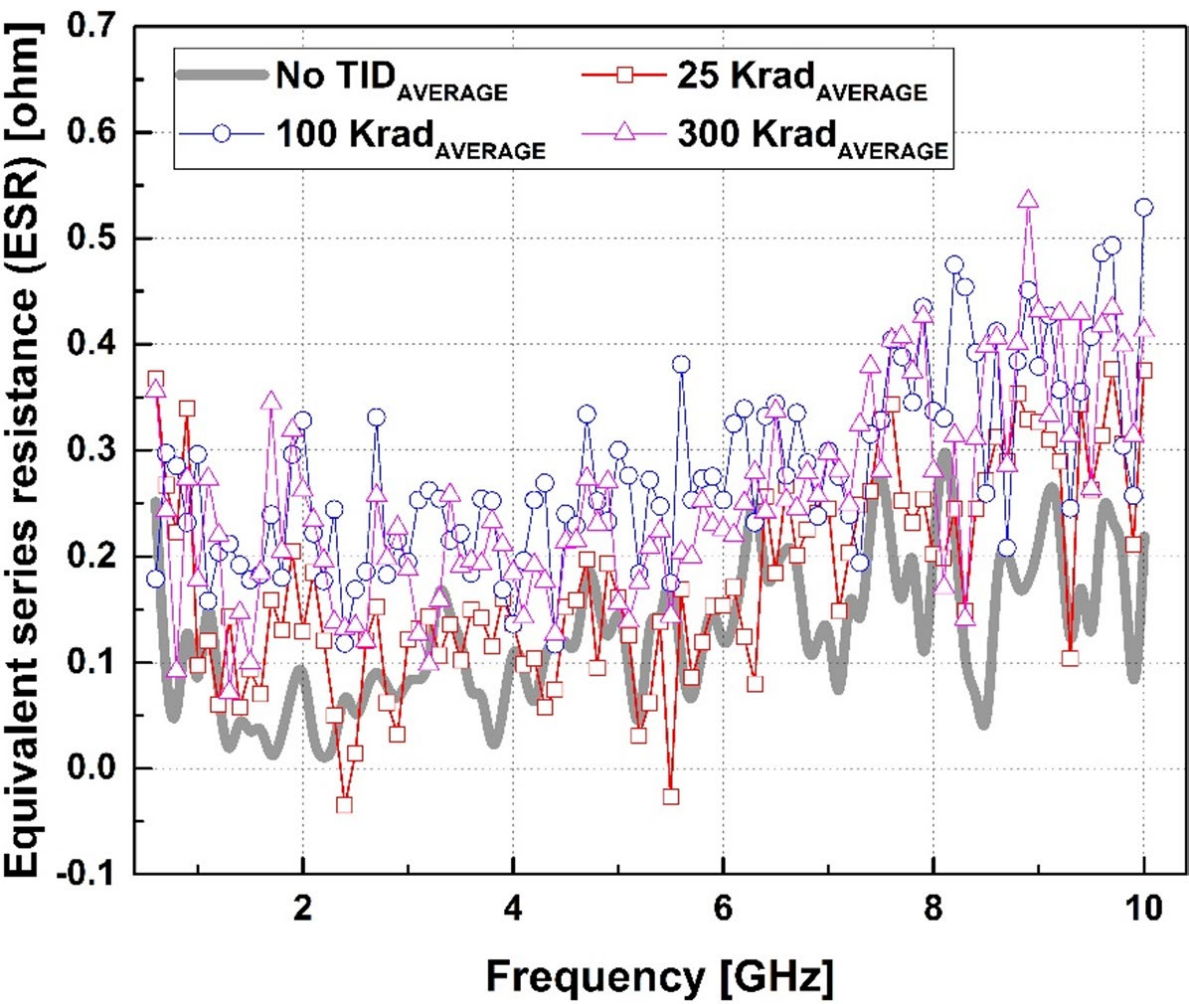
Measured ESR of the ELC for mean values under TID conditions.

Figure 2 shows the mean ESR for the total-ionizing-dose conditions. Across the measured frequency range up to 20 GHz, the ESR remained small and showed only minor variation with dose. At 10 GHz, the ESR variation across samples ranged from − 0.24 to + 0.27 Ω at 25 krad(Si), − 0.07 to + 0.38 Ω at 100 krad(Si), and − 0.16 to + 0.39 Ω at 300 krad(Si). Within the evaluated dose range, the ESR increased slightly by approximately 0.1–0.2 Ω with total ionizing dose.

### Inductance and quality factor of the spiral Inductors

The effective inductance of the spiral inductor was extracted from the de-embedded input admittance based on the imaginary component of the measured input admittance.

**Fig. 3:**
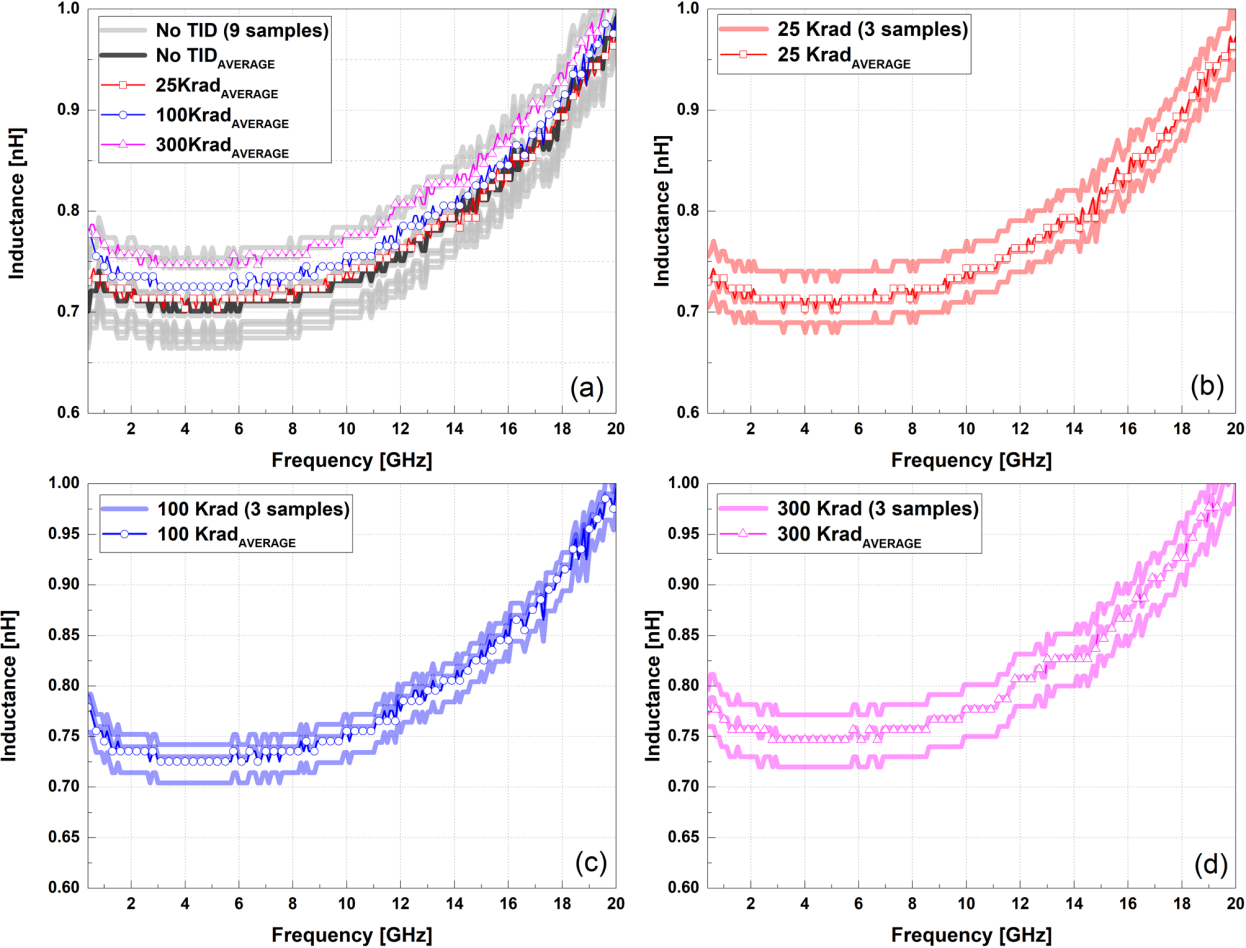
Measured inductance of the spiral inductor for nine samples and mean values under four TID conditions.

Figure 3 summarizes the inductance measured from 9 samples for the total-ionizing-dose conditions. In subfigure (a), the 9 samples at 0 krad(Si) are shown together with the mean inductance values obtained at 25, 100, and 300 krad(Si). Subfigures (b)–(d) present the three samples and the corresponding mean value for each dose level.

Across the measured frequency range up to 20 GHz, the extracted inductance values remained similar for all dose conditions. The mean inductance values are 0.77 nH at 0 krad(Si), 0.78 nH at 25 krad(Si), 0.79 nH at 100 krad(Si), and 0.81 nH at 300 krad(Si).

**Fig. 4:**
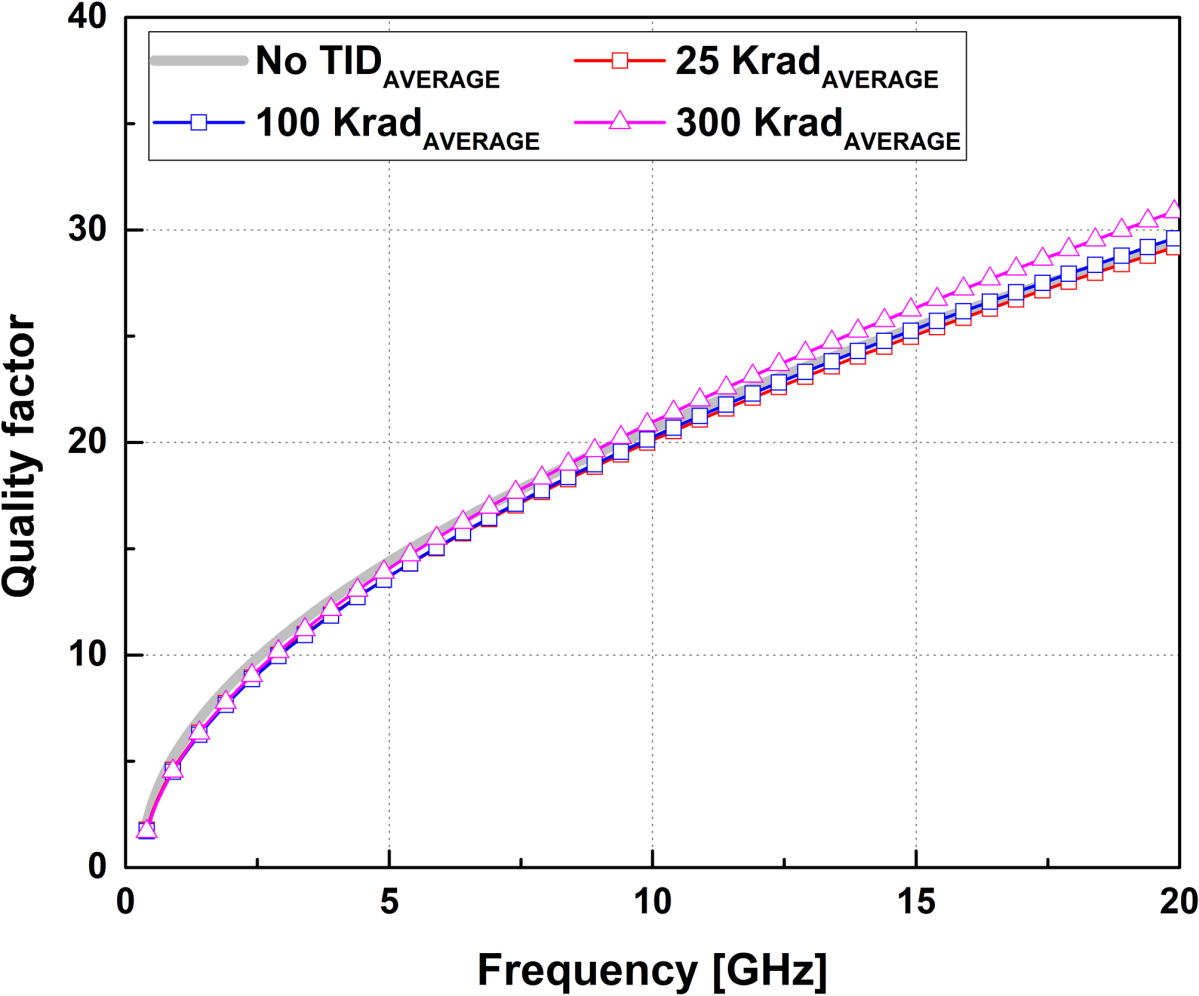
Mean Q-factor of the spiral inductor with power-root trend fitting applied.

The mean quality factor (Q-factor) of the spiral inductor was extracted from the de-embedded input parameters based on the ratio between the reactive and resistive components of the measured impedance. Figure 4 presents the mean Q-factor of the spiral inductor under the four TID conditions. The Q-factor increases with frequency across the measured band up to 20 GHz, and the differences among the dose conditions remain small. To reduce fluctuation at higher frequencies, a power-root trend fitting was applied for averaging. Representative values of the extracted inductance and Q-factor at 2 GHz and 10 GHz are summarized in Table 2.

**Table 2:** Extracted mean inductance and sample variation at a representative frequency.

Dose (Krad)	Mean L_eff_ + σ @2GHz [nH]	Mean Q + σ @2GHz	Mean L_eff_ + σ @10GHz [nH]	Mean Q + σ @10GHz
no TID	0.72 + 0.03	8.29 + 0.03	0.73 + 0.03	19.6 + 0.03
25	0.71 + 0.03	8.32 + 0.03	0.74 + 0.03	20.1 + 0.03
100	0.74 + 0.02	7.59 + 0.02	0.76 + 0.02	21.8 + 0.02
300	0.76 + 0.03	7.93 + 0.03	0.78 + 0.03	20.3 + 0.03

### Impedance behavior of an RF matching network under TID-equivalent conditions

The impedance behavior of a representative RF input matching network was evaluated under TID-equivalent conditions by applying radiation-equivalent capacitor models derived from the measured characteristics of the edge-lift capacitor.

**Fig. 5:**
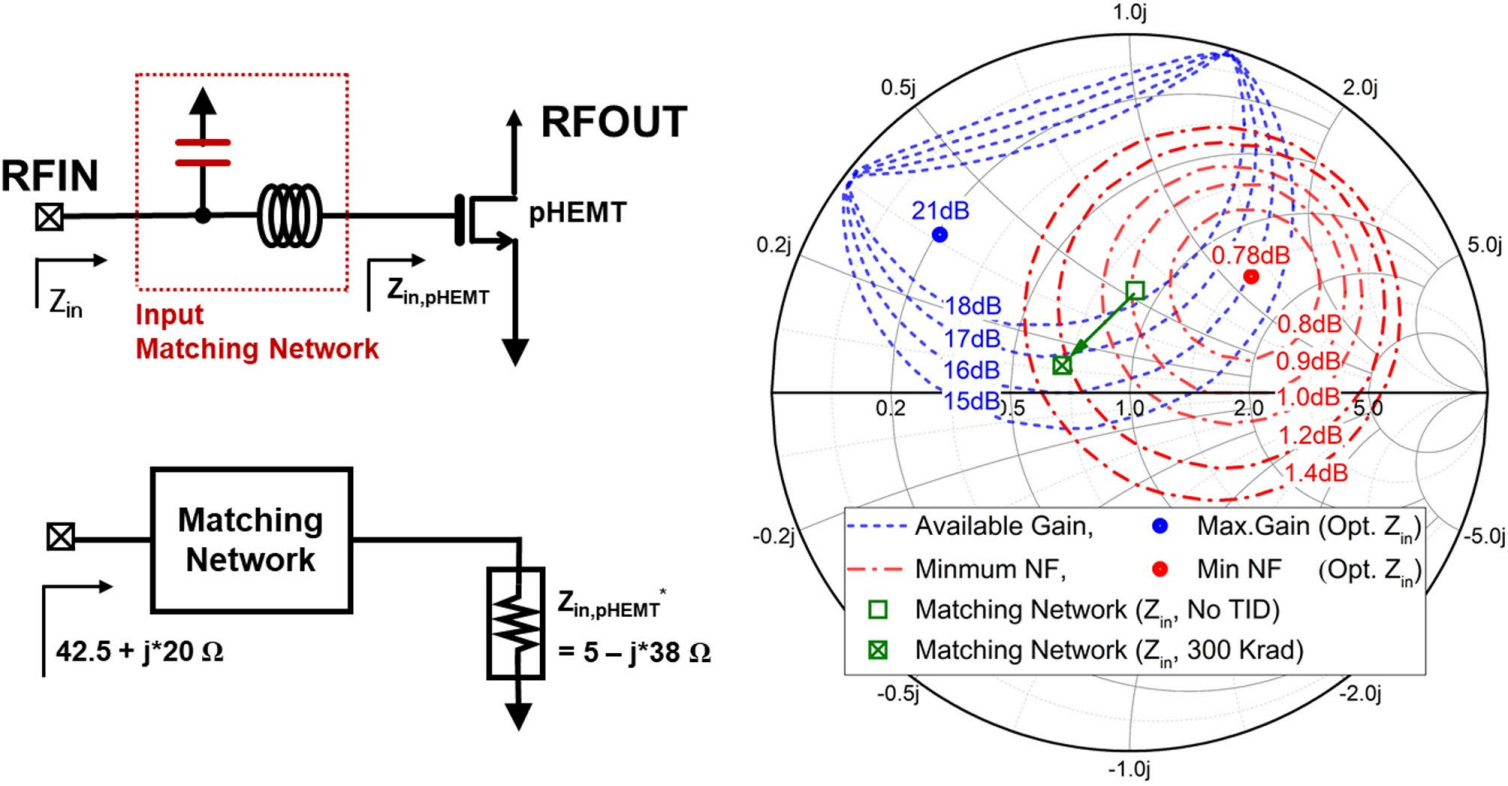
Simplified RF amplifier with an LC matching network and Smith chart showing available gain and minimum noise figure contours along with impedance trajectories under no TID and TID-equivalent capacitor models.

Figure 5 shows the Smith chart of the input impedance of the matching network at 10 GHz for the no-TID and TID-equivalent conditions. Under the no-TID condition, the input impedance is located near 43.75 + j27.15 Ω (Z_in, no TID_). When the TID-equivalent capacitor model is applied, the impedance shifts toward 33.8 + j5.35 Ω (Z_in, 300 Krad_), indicating a clear displacement of the impedance point on the Smith chart. Correspondingly, the impedance point is located near the 1.0 dB noise circle under the no-TID condition and moves toward the 1.3 dB noise circle after 300 krad irradiation. In addition, the small-signal gain decreases from 18.1 dB to 16.8 dB under the TID-equivalent condition.

**Fig. 6:**
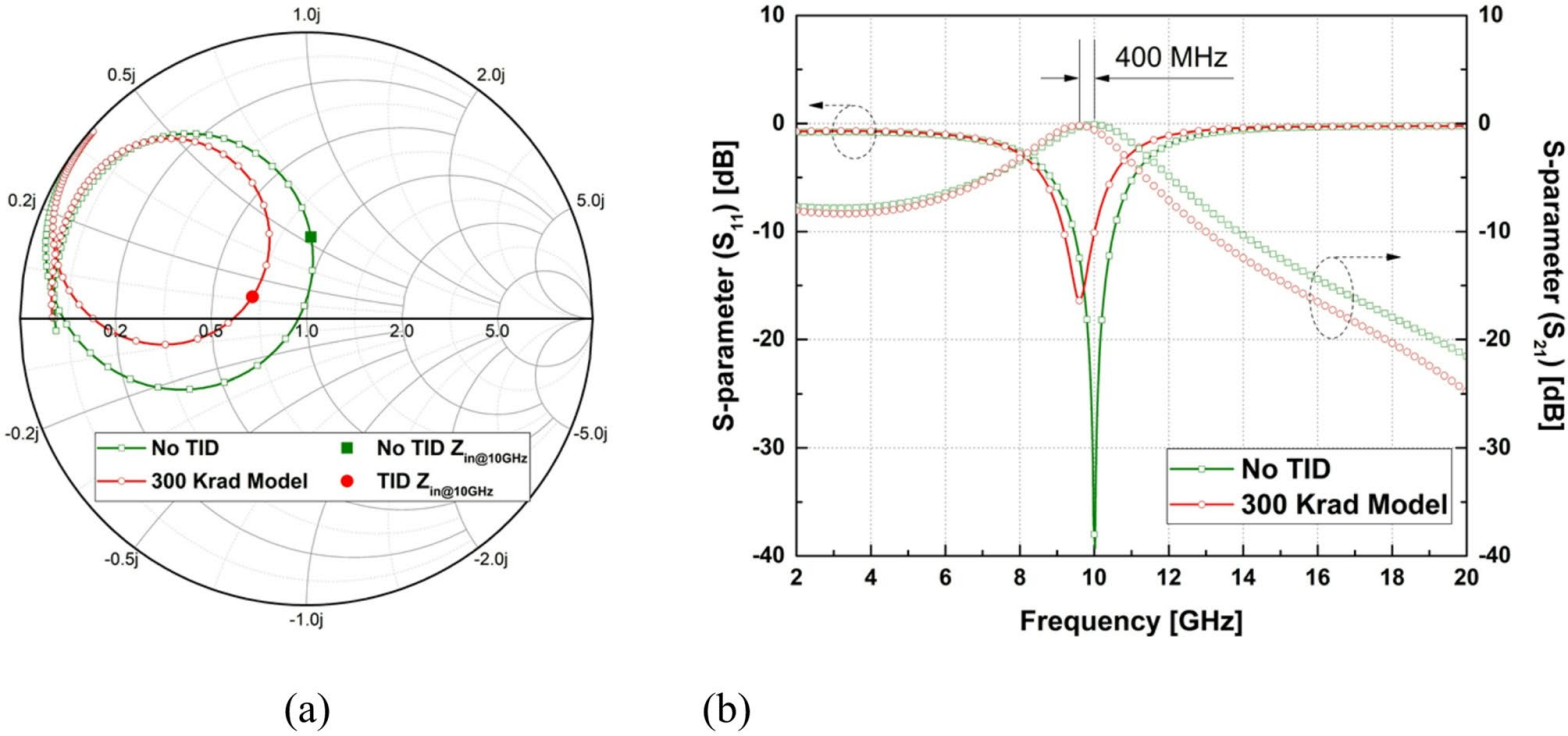
(a) Input-impedance trajectories on the Smith chart from 2 to 20 GHz under no TID and TID-equivalent conditions; (b) corresponding S₁₁ and S₂₁ responses showing frequency shift and insertion-loss degradation.

Figure 6 presents the broadband impedance trajectories and S-parameter responses of the matching network. Compared with the no-TID condition, the TID-equivalent condition results in a downward shift of the matching frequency and increased insertion loss around 10 GHz. These changes are consistently observed across the evaluated frequency range.

## Discussion

To evaluate cumulative total ionizing dose effects, a conservative accelerated test condition of 300 krad(Si) was applied in this study, under which the experimental results demonstrate that the edge-lift capacitor exhibits a markedly stronger response than other passive components fabricated in the same GaAs MMIC process. As shown in Fig. 1, the effective capacitance of the ELC increases significantly with dose, whereas Figs. 3 and 4 show that the spiral inductor exhibits only minor variations in inductance and quality factor under identical irradiation conditions. This clear contrast indicates that radiation sensitivity in GaAs passive devices is governed primarily by device geometry and electromagnetic field distribution rather than by the process platform itself.

**Fig. 7:**
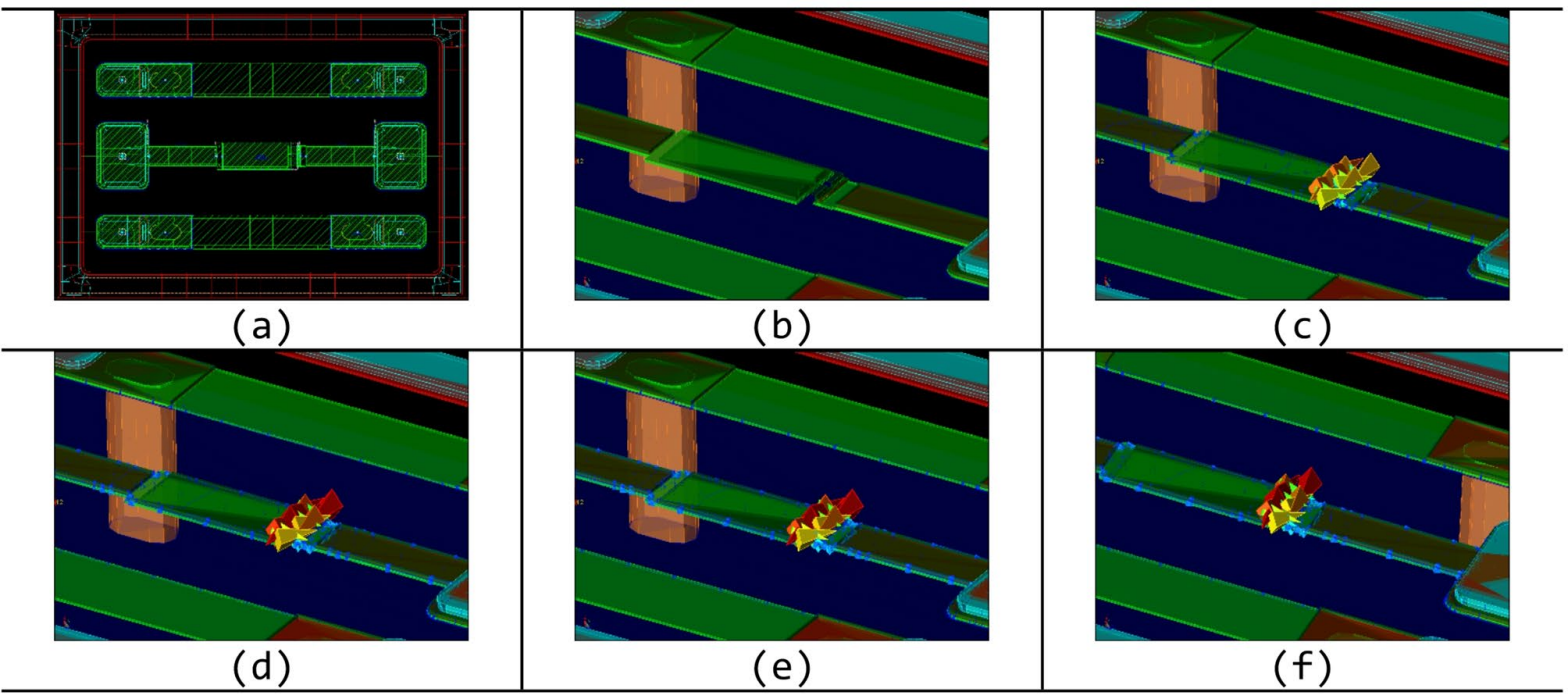
EM simulation of the ELC: (**a**) layout geometry; (**b**)–(**f**) simulated electric-field distribution near the lifted metal edge at 0 Hz, 1 GHz, 6 GHz, 11 GHz, and 20 GHz.

The pronounced capacitance increase observed in the ELC can be attributed to its fringe-field–dominant structure. As shown by the simulated electric-field distributions as a function of operation frequency in Fig. 7, a substantial portion of the electric field in the edge-lift geometry extends into the surrounding dielectric region near the lifted metal edge. As a result, the effective capacitance becomes highly sensitive to local variations in dielectric properties.

**Fig. 8:**
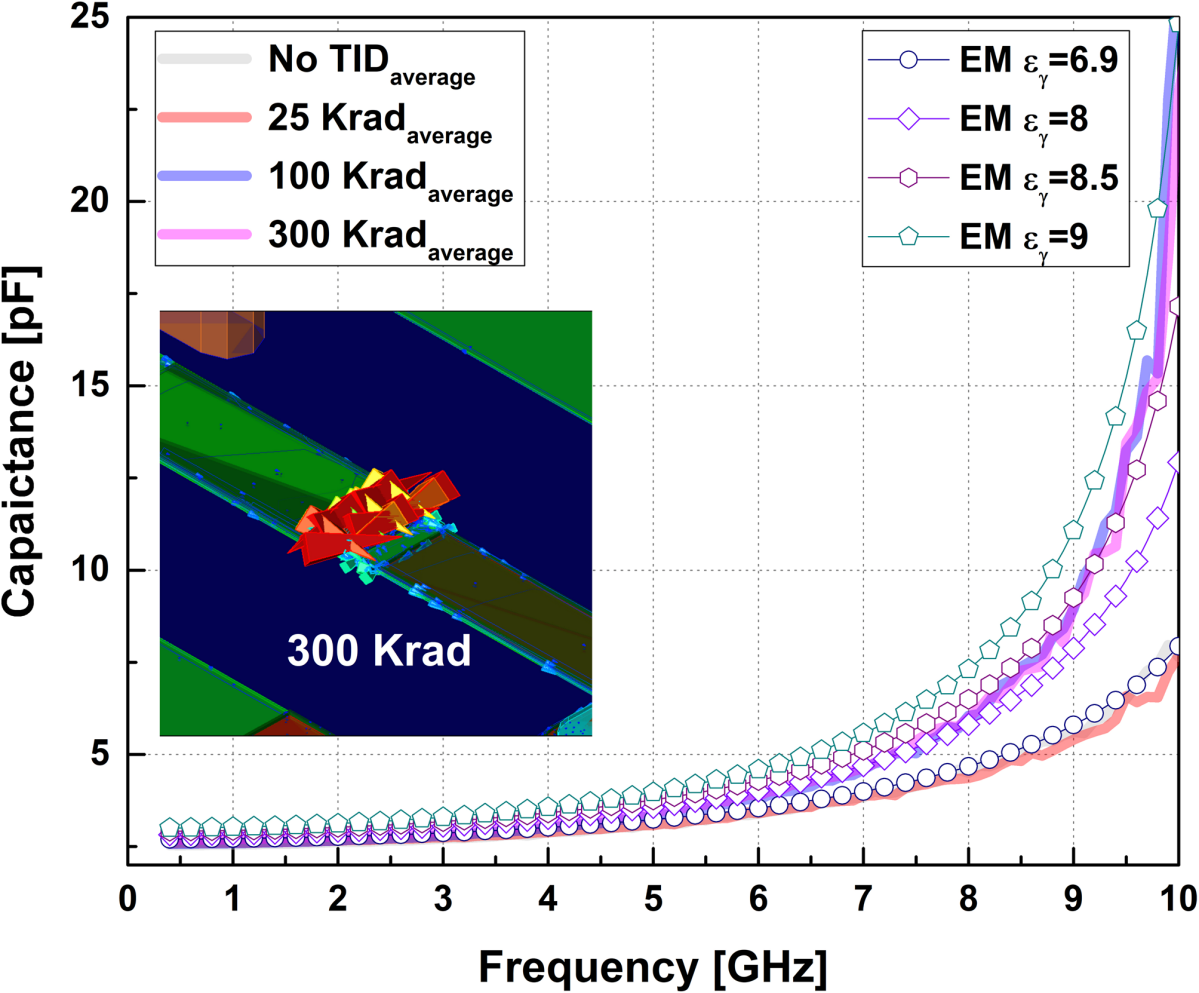
Comparison of measured effective capacitance and EM-simulated capacitance obtained from dielectric-permittivity sweep under different TID conditions.

Furthermore, Fig. 8 shows that the measured capacitance variation can be consistently reproduced by increasing, through radiation-equivalent simulations, the relative permittivity of the SiN dielectric layer participating in the fringe-field region. The extracted permittivity should therefore be interpreted as an effective modeling parameter that captures the cumulative impact of radiation-induced dielectric modification, rather than as a direct material constant. In addition, the model agreement can be further improved by selectively applying different effective permittivity values depending on the target operating frequency range of interest.

The ESR results further support this interpretation. As shown in Fig. 2, although the ELC exhibits a pronounced increase in effective capacitance with accumulated dose, the corresponding change in ESR remains small over the measured frequency range. This observation indicates that radiation-induced impedance variation in the ELC is dominated by changes in the reactive component rather than by increased resistive loss.

In contrast, the spiral inductor exhibits comparatively stable inductance and quality factor across all evaluated dose conditions. As summarized in Figs. 3 and 4, the extracted inductance and Q-factor remain nearly unchanged over the measured frequency range, reflecting the fact that the dominant electromagnetic fields in the inductor are primarily associated with current flow in the metal traces, with relatively limited interaction between the electric field and the surrounding dielectrics. As a result, radiation-induced dielectric-property variations exert only a secondary influence on the inductive characteristics.

The circuit-level implications of this behavior become evident when the radiation-equivalent capacitor model is applied to a representative RF input matching network. In Fig. 5, the Smith chart shows that, under no-TID conditions, the input impedance at 10 GHz is positioned near the intersection of the available-gain and noise-figure contours corresponding to a balanced compromise between gain and noise performance. When the capacitor is replaced with the radiation-equivalent EM model incorporating the measured capacitance increase under TID exposure, the input impedance shifts toward a more capacitive region on the Smith chart, increasing the distance from both the optimum gain and optimum noise contours. As further illustrated in Fig. 6, this impedance displacement leads to a downward shift of the matching frequency and degradation of the S-parameter response, including reduced available gain and increased noise figure.

These observations highlight that radiation-induced parameter variation in fringe-field–dominant passive components can propagate directly into RF performance degradation at the circuit level, even when the active device itself is assumed to remain unaffected. Conventional PDK-based electromagnetic models that assume fixed dielectric properties may therefore fail to capture critical radiation effects in impedance-sensitive RF circuits. Incorporating radiation-aware passive-device modeling during the design stage is essential for improving the robustness and performance predictability of GaAs RF front-end circuits intended for radiation environments.

## Conclusion

This study analyzed the total ionizing dose (TID) response of passive components fabricated in a GaAs MMIC process, with emphasis on fringe-field–dominant structures. Experimental results confirmed that the edge-lift capacitor exhibits a pronounced increase in effective capacitance under TID exposure, whereas the spiral inductor maintains relatively stable inductance and quality factor even under identical irradiation conditions. By applying a radiation-equivalent dielectric model, the measured capacitance variation was quantitatively correlated with impedance detuning in an RF input matching network.

The results of this work should be interpreted as an experimental case study demonstrating how radiation-induced dielectric property changes in fringe-field–dominant passive components can propagate into circuit-level RF performance variations. Such TID effects are expected to be particularly relevant in satellite and spaceborne electronic systems, which may be exposed to accumulated ionizing doses ranging from several hundred kilorad to approximately 1 Mrad depending on orbital environment and long-term mission duration. In this context, the present study provides practical insight into the radiation-environment behavior of GaAs MMIC passive components and impedance-sensitive RF circuits.

## Methods

### Device structures and layouts

**Fig. 9:**
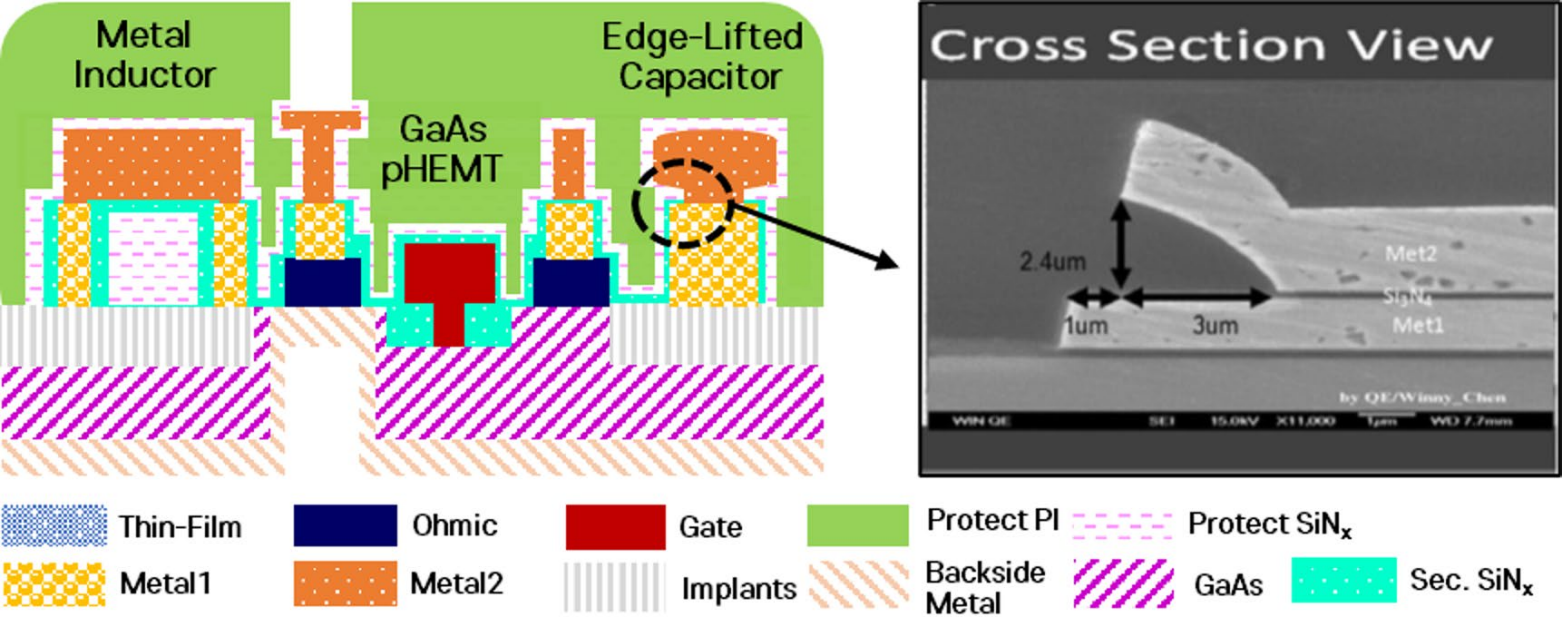
Schematic cross-section of the GaAs passive device structures and SEM image of the edge-lift capacitor^12^.

The passive devices investigated in this study were fabricated using a commercial GaAs MMIC process. Figure 9 shows the cross-sectional structures of the edge-lift capacitor and the spiral inductor evaluated in this work. The ELC adopts a metal–insulator–metal configuration in which the upper metal layer is partially lifted from the dielectric surface, forming an edge-lift structure. This geometry results in a strong fringe electric field concentrated near the lifted metal edge, which plays a critical role in determining the effective capacitance of the device. The dielectric layer between the metal electrodes consists of silicon nitride (SiN).

The spiral inductor was implemented using multi-turn metal traces with defined line width and spacing. The inductor structure was designed to ensure compatibility with the same GaAs MMIC process used for the ELC while maintaining stable RF characteristics. The nominal geometrical parameters of the edge-lift capacitor and the spiral inductor used in this study are summarized in Table 3.

**Table 3:** Key dimensions and nominal parameters of the evaluated passive devices.

Material	Thickness (*μm*)	Permittivity (E_*r*_)
GaAs	100	12.9
SiN	0.15	6.9
Passivation (Protect PI)	2.3	2.9
Protect SiN	0.3	6.9

### Total-ionizing dose irradiation conditions

**Fig. 10:**
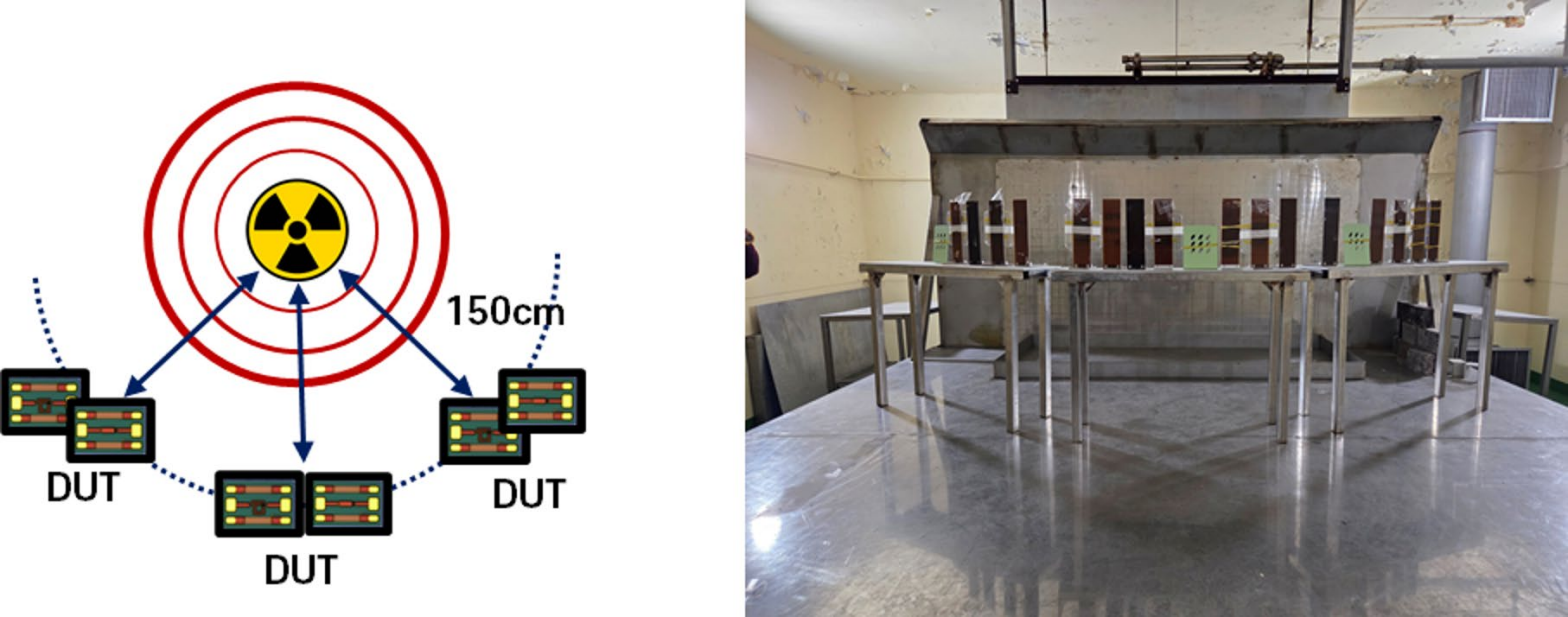
Gamma-ray irradiation facility and test environment.

The total-ionizing-dose irradiation was conducted at a gamma-ray irradiation facility using a Co-60 source at the Advanced Radiation Technology Institute in South Korea. The devices were placed on the irradiation table at a fixed distance of 150 cm from the source. The accumulated dose levels were set to 25, 100, and 300 krad(Si). All samples were irradiated under unbiased conditions to exclude bias-dependent charging effects. The devices were mounted on a low-loss fixture to maintain stable positioning during exposure. A schematic illustration of the irradiation environment is shown in Fig. 10.

### Chip-level measurement and parameter extraction

**Fig. 11:**
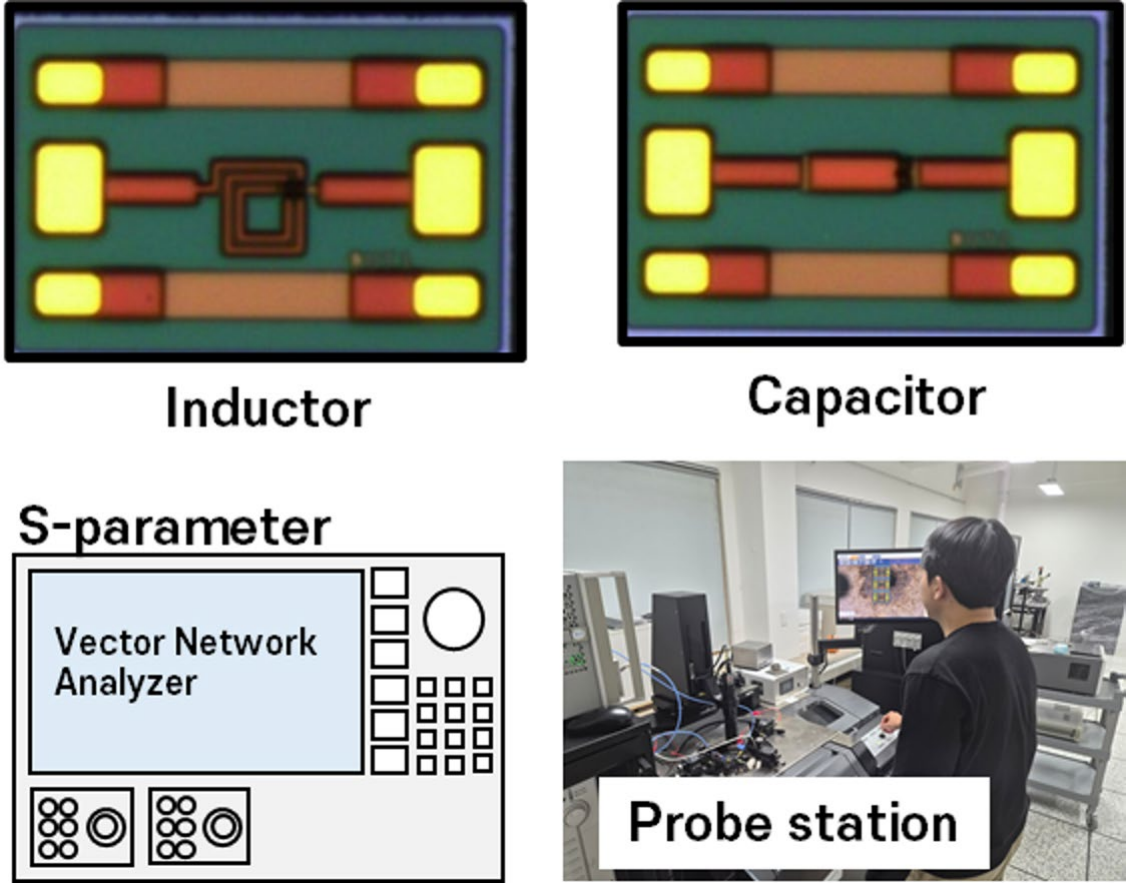
Chip-level S-parameter measurement environment.

S-parameter measurements were performed on sawn-chip samples using a vector network analyzer over the 0.1–20 GHz frequency range. After γ-ray irradiation, all devices were allowed to stabilize at room temperature for approximately 7 days prior to S-parameter measurements in order to minimize short-term transient charging effects. No intentional thermal annealing was applied during this period, and all measurements were performed under identical post-irradiation conditions. Ground–signal–ground (GSG) probes were used to contact the on-chip pads. Prior to measurement, the vector network analyzer was calibrated up to 20 GHz using an auto-calibration kit provided with the instrument. The measurement system was calibrated using a DUT–OPEN–SHORT procedure, and open and short de-embedding structures fabricated in the same process were used to remove pad and interconnect parasitics. Specifically, a two-step de-embedding procedure following the method described in [14] was applied, in which the open structure was first used to remove pad-related parasitic admittances, followed by the short structure to account for series parasitic effects associated with interconnects and contacts. The overall measurement configuration is illustrated in Fig. 11.

The effective capacitance and equivalent series resistance of the edge-lift capacitor were extracted from the de-embedded admittance and impedance parameters. The effective capacitance was calculated from the imaginary part of the input admittance, and the ESR was obtained from the real part of the input impedance, as given by

1 $${C}_{eff}=\frac{Im\left({Y}_{11}\right)}{\omega}$$

2 $$ESR=Re\left({Z}_{11}\right)$$

The effective inductance and quality factor of the spiral inductor were obtained from the de-embedded input parameters using

3 $$L_{{eff}} = - \frac{1}{{\omega \mathrm{Im} \left( {Y_{{11}} } \right)}}$$

4 $$Q = \frac{{\omega L_{{eff}} }}{{R_{{eff}} }}$$

The same extraction procedure was consistently applied to the pre-irradiation (no TID) condition and to all irradiated conditions of 25, 100, and 300 krad(Si).

### EM simulation and radiation-equivalent modeling

**Fig. 12:**
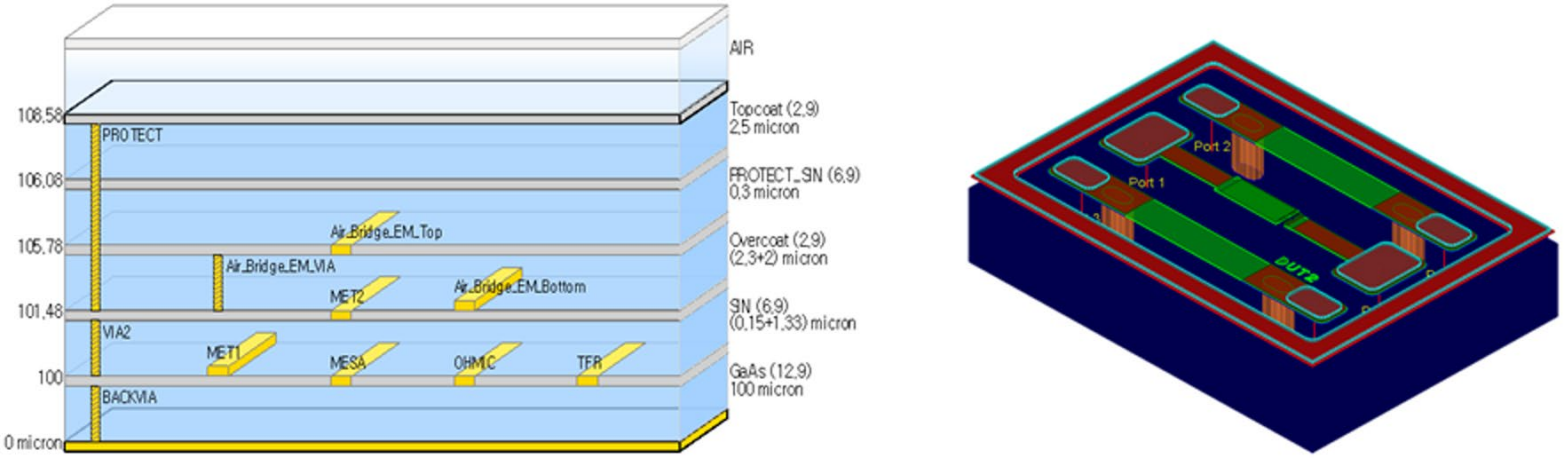
Process stack representation and 3D layout geometry used for EM simulations under nominal process conditions.

Electromagnetic simulations were performed using a commercial 3D EM solver under nominal process conditions. The simulation models incorporated both the process stack representation and the three-dimensional layout geometry provided by the GaAs MMIC process, as shown in Fig. 12. For the evaluation of equivalent dielectric constant (ELC) variations, the ADS simulation software was employed, and the EM simulation setup was established using a standard foundry-provided PDK supporting the GaAs process. The baseline EM simulation was conducted using the default process parameters defined in the PDK. Table III summarizes the nominal thickness and permittivity values provided by the foundry, which were directly applied in the simulations. In the EM simulations, the ports were defined using the default DIRECT FEED configuration, and the frequency range was swept from 1 to 20 GHz.

For radiation-equivalent modeling, the relative permittivity of the dielectric layers was swept around the nominal process values. Preliminary simulations confirmed that variations in the permittivity of the passivation SiN layers produced negligible changes in the extracted capacitance. Accordingly, the permittivity sweep was applied only to the SiN dielectric located between the MET_1_ and MET_2_ layers, which directly participates in the fringe-field region of the edge-lift capacitor, while all other stack parameters were kept at their nominal values. At each permittivity condition, the simulated impedance was used to extract capacitance and inductance values using the same equations applied to the measured data.

## Data Availability

The data that support the findings of this study are available from the corresponding author upon reasonable request.
